# Molecular characterization of sequence-driven peptide glycation

**DOI:** 10.1038/s41598-021-92413-7

**Published:** 2021-06-24

**Authors:** Michelle T. Berger, Daniel Hemmler, Alesia Walker, Michael Rychlik, James W. Marshall, Philippe Schmitt-Kopplin

**Affiliations:** 1 Chair of Analytical Food Chemistry, Technical University Munich, Maximus-von-Imhof-Forum 2, 85354 Freising, Germany; 2 Research Unit Analytical BioGeoChemistry (BGC), Helmholtz Zentrum München, Ingolstädter Landstrasse 1, 85764 Neuherberg, Germany; 3 The Waltham Pet Science Institute, Mars Petcare UK, Waltham-on-the-Wolds, Leicestershire, LE14 4RT UK

**Keywords:** Mass spectrometry, Peptides, Monosaccharides, Chemical modification

## Abstract

Peptide glycation is an important, yet poorly understood reaction not only found in food but also in biological systems. The enormous heterogeneity of peptides and the complexity of glycation reactions impeded large-scale analysis of peptide derived glycation products and to understand both the contributing factors and how this affects the biological activity of peptides. Analyzing time-resolved Amadori product formation, we here explored site-specific glycation for 264 peptides. Intensity profiling together with in-depth computational sequence deconvolution resolved differences in peptide glycation based on microheterogeneity and revealed particularly reactive peptide collectives. These peptides feature potentially important sequence patterns that appear in several established bio- and sensory-active peptides from independent sources, which suggests that our approach serves system-wide applicability. We generated a pattern peptide map and propose that in peptide glycation the herein identified molecular checkpoints can be used as indication of sequence reactivity.

## Introduction

Glycation presents a ubiquitous non-enzymatic post-translational modification^1,2^, which is formed by the reaction of amino compounds and reducing sugars. It refers to a complex reaction network and produces a multitude of heterogeneous reaction products, also known as Maillard reaction products (MRPs) or advanced glycation end products (AGEs)^3,4^. Glycation is a multifactorial reaction, which depends on the nature of the precursors and the reaction conditions, including time and concentration^5–8^. The Maillard reaction (MR) is one of the most common and essential reactions in food processing and determines color, flavor and taste of food. Further, its reaction products are known to affect human health^9,10^, and contribute to various pathologies, such as diabetes^11,12^. Here, glycation leads to molecular and cellular changes in a series of complicated events. Hyperglycemia drives glycation of lipids and proteins and development of vascular lesions by AGE engagement of the receptor for AGE (RAGE) in cells of the vessel wall^13–16^. Due to their broad relevance, thorough understanding of glycation reactions is indispensable.

As insights into the MR of amino acids continue to emerge, new models are needed to improve the understanding of peptide and protein glycation^17^. Many previous studies analyzing the health effects of glycation products and peptides point to their miscellaneous bioactivities and their potential as nutraceuticals and functional food ingredients^18–23^. Glycation induced alterations in the bioactivity and improvement of sensory attributes have been described for various peptide mixtures^24–32^. However, the specific peptides related to these changes remain largely uncharacterized and, even more important, the behavior of peptides in glycation reactions has barely been systematically analyzed and thus is unaccounted. Only a limited number of studies on peptide reactivity in the MR have been conducted and focused on synthetic peptides^26,33,34^ or peptide derived MRPs in specific foods^35–38^. These approaches have revealed the relevance of both peptide length and composition in the MR and the importance of peptide glycation in various fields, not only including diverse food matrices but also biological systems and disease progression^10^. The information describing the general determinant factors of peptide glycation, however, remains elusive. Therefore, particular sequences and, thus classes of proteins that have preference to undergo glycation reactions and the final consequences, such as loss and gain of bioactivities, cannot be determined. Especially required are model systems for large-scale MRP characterization that enable general understanding of the influence of the amino acid composition, sequence and peptide length on glycation product formation.

To acquire a better understanding of site-specific peptide glycation the analysis of the Amadori product (AP), a relatively stable intermediate of the MR, and consecutive downstream reaction products MRPs is particularly well-suited. High-resolution mass spectrometry (MS) is a fast and highly sensitive method, which enables detection and identification of both early and advanced products of the MR^39,40^. Information on net chemical transformations and precursor reactivity in such systems can be gained by non-targeted experiments and generation of mass difference networks^41,42^. Collision-induced dissociation (CID) after electrospray ionization (ESI) MS has been successfully applied for peptide derived AP analysis^43–45^. However, non-targeted large-scale analysis and interpretation of peptide derived MRPs is limited. Only a few studies have gained insight into peptide reactivity and the influence of sequence microheterogeneity^33,34,46–48^. This includes glycation based on accessibility of the N-terminus and catalytic effects in some short-chain peptides. Here, we report that the combination of high-resolution ESI quadrupole time of flight (QTOF) MS, bioinformatics and multivariate statistics enables a deep and molecular-level investigation of complex peptide systems. Using this combinatorial method for large-scale AP analysis, we characterized the reaction behavior of 264 casein-derived peptides in the MR and used this data to gain insight into sequence-dependent differences in AP formation profiles and, thus, peptide reactivity. Furthermore, we discovered potentially relevant glycation-patterns and demonstrate system-wide applicability of this study to various food-peptide sources by in silico sequence mapping. Database search serves as a reference for investigation of bioactive and sensory-active peptide reaction behavior. This approach may be amendable to practically any type of glycation system, and it allows exploration at various levels of information, from the influence of the peptide composition to the role of specific sequence-patterns in peptide glycation.

## Results

### A time-resolved analysis of peptide dependent Amadori product formation

**Figure 1:**
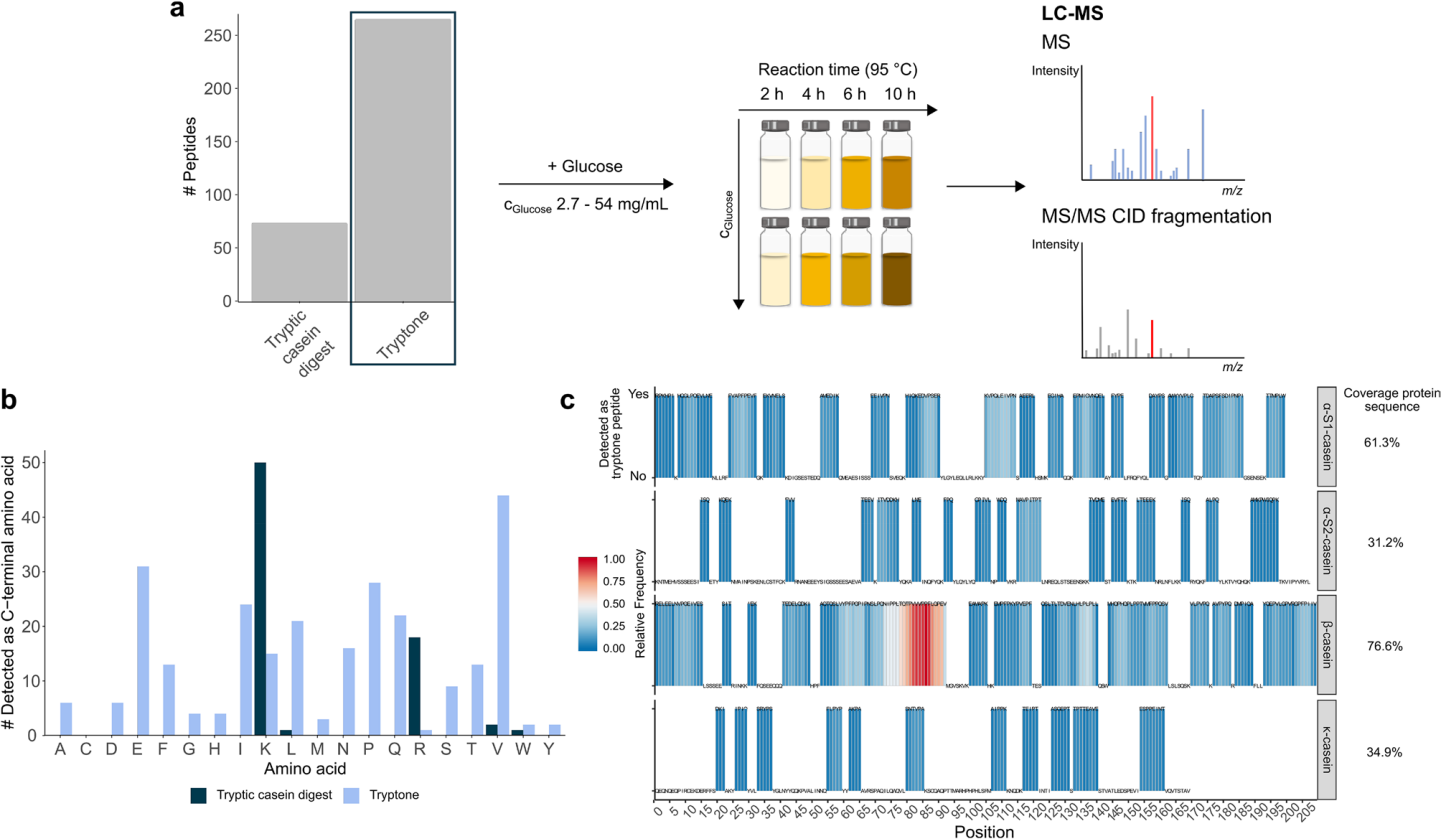
Analysis of tryptone glucose model systems by UHPLC-QTOF-MS. **(a)** Experimental design—high-resolution mass spectrometry was used to analyze tryptone glucose model systems and for the identification of site-specific non-enzymatic glycation. (**a)** The bar plot illustrates the number of peptides provided by an in silico tryptic casein digest (72) compared to tryptone (264). (**b)** A bar plot shows the number of C-terminal amino acids observed for tryptone peptides (light blue) and a theoretical casein digest by trypsin (dark blue). Tryptic digestion predominantly forms peptides with C-terminal lysine or arginine. (**c)** A casein protein heatmap represents how often (relative scale) detected tryptone peptides covered the same amino acid sequence in the proteins, showing which protein substructures contribute to peptide heterogeneity of the model systems. Dipeptides were removed for more sequence specificity.

To study the reaction behavior of peptides in glycation, we heated complex model systems containing glucose (2.7–54 mg/mL) and tryptone at 95 °C for 2, 4, 6 and 10 h, respectively. Compared to an in silico tryptic protein digest, tryptone provides approximately four times more free peptides with increased diversity (Fig. 1a). A vast number of non-enzymatic cleavage sites generates many diverse peptides (Supplementary Fig. 1) with partially overlapping amino acid sequences. This enables characterization of site-specific microheterogeneity and, ultimately, identification of specific sequence patterns that promote glycation. Apart from that, C- and N-terminal amino acids in tryptone peptides comprise a much greater diversity than enzymatic digests can cause, which becomes apparent by comparison with an in silico tryptic casein digest (Fig. 1b and Supplementary Fig. 2a). Unlike tryptone, there is a bias toward lysine- (Lys) and arginine- (Arg) containing peptides for tryptic casein digestion (Supplementary Fig. 2b) and poor enzymatic cleavage of certain protein regions (Supplementary Fig. 3) may lead to the preclusion of particularly relevant peptides. Even alternative proteases or sequential digestion would only provide a marginally increased total number and diversity of peptides compared to trypsin^49^. With the applied method, we could nearly completely cover the casein protein sequences (Fig. 1c).

**Figure 2:**
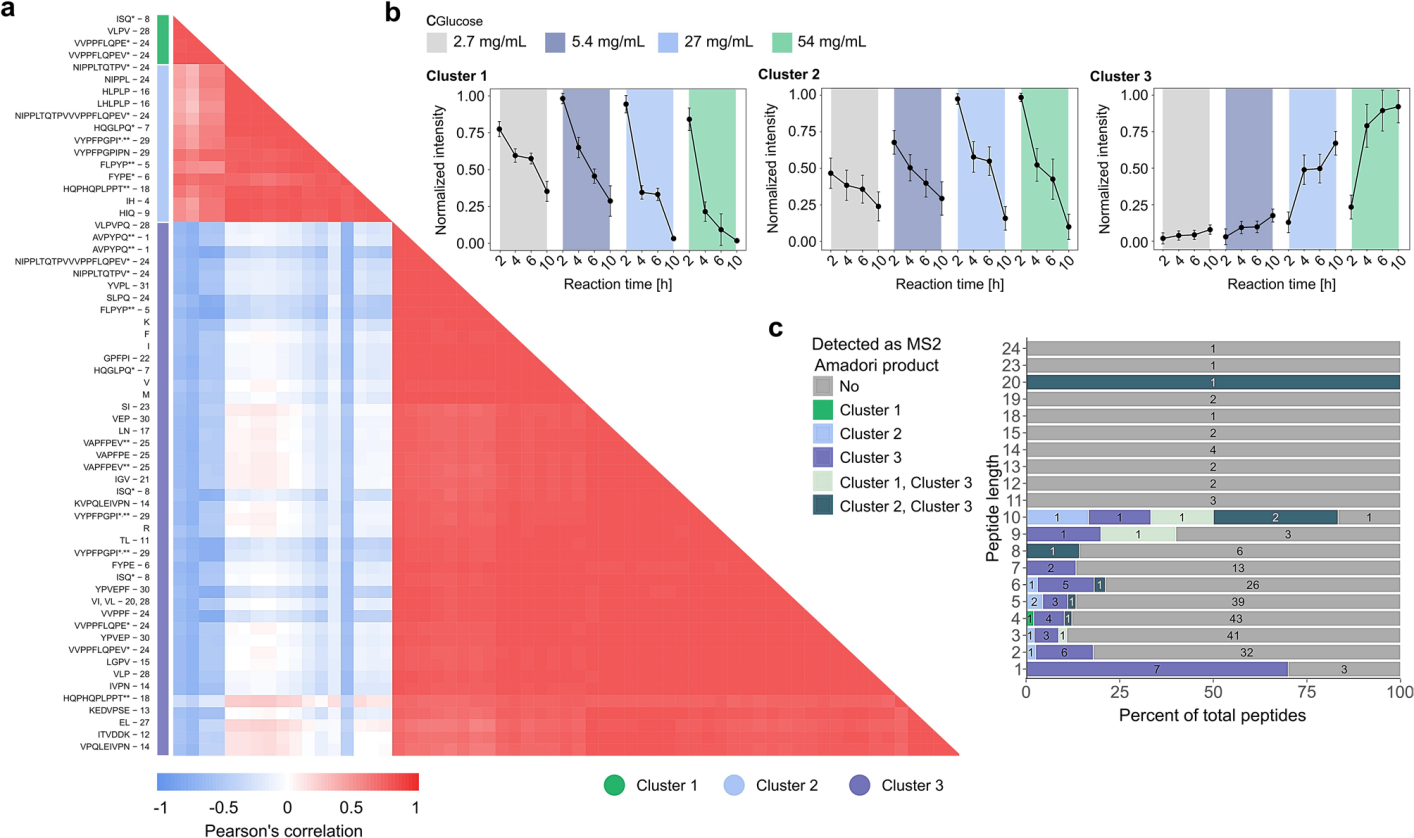
Intensity profiling enables classification of APs identified. **(a)** Pearson’s correlation heatmap computed for the intensity profiles from the APs confirmed by MS/MS fragmentation pattern. Rows are ordered by unsupervised hierarchical clustering. Positive values (red) represent higher similarity in formation/degradation rates. Negative correlation values are colored in blue. Asterisks denote APs from the same peptide (* isomers; ** different charge states). Suspended numbers indicate peptides with similar amino acid sequences. (**b)** Intensity profiles visualize time-resolved AP formation depending on the glucose concentration. Intensity values were normalized towards the greatest intensity value. Colors indicate different glucose concentrations. (**c)** A stacked bar plot shows the percentage of the total number of peptides of a certain length that were identified as an AP (colored bars). Different colors provide information on the assigned clusters as identified in **(a)**.

With this extensive dataset in hand we first explored the concentration- and time-dependent reaction behavior of this diverse pool of peptides based on the formation of the corresponding APs. The AP is a relatively stable intermediate of the early MR^50^, which is formed via condensation between the amino compound and the reducing sugar, and subsequent rearrangement^11^. The number of hexose residues coupled to an amino acid or peptide was estimated by a mass increase of 162.0528 Da per attached monosaccharide (C_6_H_12_O_6_-H_2_O). Tandem MS was applied to obtain structural information, which allowed confirmation of the AP chemical structure of 47 amino compounds (Supplementary Table 1). Significant correlations (p-value ≤ 0.05) were observed across normalized AP intensity profiles, and APs clustered by the influence of sugar concentration but also reaction time (Fig. 2a). Interestingly, APs clustered that were formed from peptides with comparable amino acid sequences. Sequence similarity is highlighted by the suspended numbers indicating sequence groups, e.g. HLPLP and LHLPLP (Cluster 2, sequence group 16). Certain APs found in Cluster 1 and 2 seemed to form isomers, which caused them to also appear in Cluster 3. In Fig. 2b, the representative normalized mean intensity profiles for the three clusters are shown, and Supplementary Fig. 4 provides the individual normalized intensity curves for each AP, separately. The intensities of APs in Cluster 1 and 2 reached their maximum after two hours for all glucose concentrations and decreased with further reaction time (Fig. 2b). In contrast, Cluster 3 contains APs, which increased with time and reached maximum intensity after either six or ten hours of incubation. Further, enrichment of AP levels at different glucose concentrations was observed. The highest intensity of Cluster 1-APs was either detected at 5.4 or 27 mg/mL of glucose, whereas for APs in Cluster 2 (27–54 mg/mL) and 3 (54 mg/mL) higher sugar concentrations were required to reach their maximum. Figure 2c compares AP formation between different peptide lengths, demonstrating that nearly all amino acids and dipeptides were found in Cluster 3, whereas larger peptides did not show uniform normalized AP intensity profiles. Moreover, the percentage observed as an AP for each peptide length is displayed, but no general correlation between peptide reactivity and sequence length could be observed. Note, this calculation is based on the total number of peptides per length, so one length may appear to be glycated to a greater extent if a minor number of peptides was identified with this length. A higher proportion of APs derived from dipeptides compared to tripeptides resembles observations from previous studies^33,43^, which suggested decreasing reactivity with increasing peptide length. Here, we confirm and extend these observations by investigating a larger range of peptide chain lengths. Interestingly, longer-chain peptide sequences were not generally associated with reduced reactivity in early glycation reactions, e.g. when comparing penta- and hexapeptides or nona- and decapeptides. Even with these observations, it is difficult to comment on the influence of peptide length on glycation on a universal level.

All APs significantly increased after 2 h (t-test, p-value ≤ 0.05); however, different glucose levels were required (Supplementary Table 1). Most (25) of the identified APs significantly increased at a glucose concentration of 2.7 mg/mL, while other peptides required higher glucose levels. Interestingly, different observations were made for highly similar peptides. For example, VPQLEIVPN required a glucose concentration of 27 mg/mL to increase, significantly, whereas for KVPQLEIVPN only 5.4 mg/mL of glucose were needed. Analogous behavior was observed for the APs of VAPFPE (27 mg/mL) and VAPFPEV (5.4 mg/mL).

### Shedding light onto the role of peptide composition in glycation

**Figure 3:**
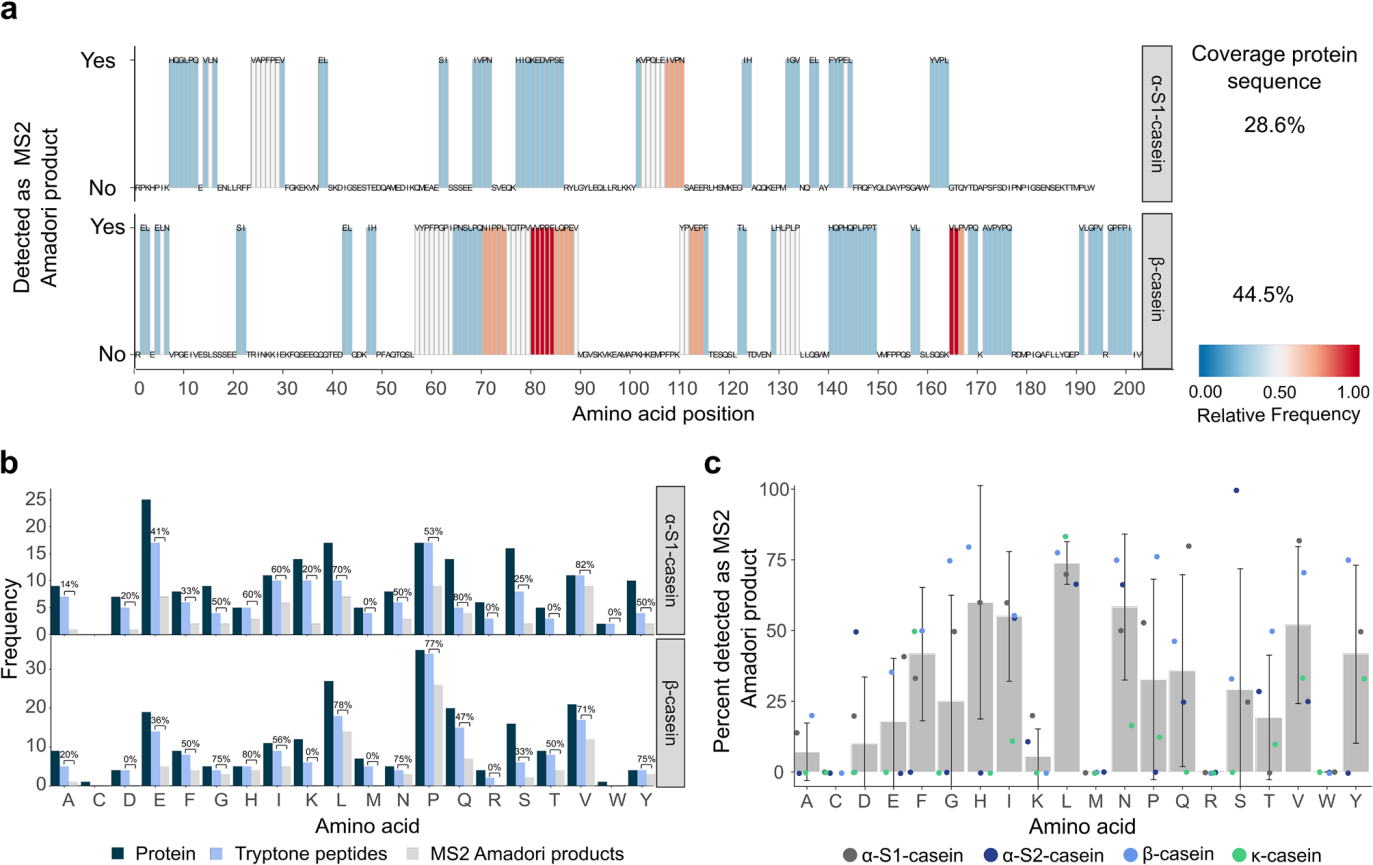
Deconvolution of glycated protein sequences reveals their compositional characteristics.** (a)** Contribution of protein regions and the amino acid microenvironment to peptide AP formation. Casein protein heatmaps represent the relative frequency of amino acid positions that were detected as an AP, indicating which peptides from specific protein regions contribute most to early MR and how the amino acid microenvironment influences peptide reaction behavior. Approximately 28.6% of α-S1- and 44.5% of β-casein were detected as the corresponding MS2 AP, and the majority contains redundant sequence motifs (i.e. P-E-V, see Fig. 4). (**b)** Abundance of amino acids in casein proteins (dark blue), tryptone peptides (light blue) and APs (gray). Embedded values indicate the percentage of peptides that could also be detected as an AP. (**c)** The bar plot depicts the median percentage of the amino acid found in APs in the different casein proteins. Overlaid points indicate the percentage for each studied casein protein. The whiskers represent the standard deviation.

AP analysis uniquely facilitates characterization of site-specific glycation, and our dataset provides insight into the highly complex and yet largely unknown reaction behavior of peptides in glycation. Previous studies explored peptide derived MRPs in particular foods providing limited information from a global prospect^35–38^. Others have investigated the reactivity of highly specific synthetic peptides depending on factors explored herein to some degree, such as peptide length (discussed above) and amino acid composition^33,34^. Here, we aspired to approach these research questions from a general level using a large reservoir of peptides and APs. Mapping glycated peptides onto casein proteins revealed that AP formation was observed for only 14% of α-S2- and 9% of κ-casein peptides (Supplementary Fig. 5), but APs related to α-S1-casein (29%) and ß-casein (45%) were detected to a larger extent (Fig. 3a). Analyzing the amino acid sequence of α-S2- and κ-casein peptide APs, we identified protein-specific peptides such as FLPYP (F_55_-P_59_ of κ-casein) . The ability to profile glycosites at this scale provides opportunities to determine the relative susceptibility of peptide collectives with similar amino acid sequences to the early MR. Especially reactive peptide classes are captured here, as the protein heatmaps show the frequency that sequences co-occurred on glycated peptides . AP formation of peptides sharing certain amino acid sequences appeared to be favored, e.g. including peptides that originated from N_73_–V_92_ and V_170_–V_173_ of β-casein.

To examine the influence of the amino acid composition on peptide reactivity in the early phase of glycation, we calculated the contribution of each amino acid to AP formation, given as a percentage of the total observations in tryptone peptides. Figure 3b shows amino acids, e.g. glutamic acid (Glu) and leucine (Leu), that appeared equally in peptide APs from α-S1- (top) and β-casein (bottom). Other amino acids, such as proline (Pro) and histidine (His) showed considerable variations in their contribution to AP formation (Supplementary Fig. 6) depending on the source protein*,* meaning the overall peptide sequence, which fits with the known role of the microenvironment of amino acids in glycation. Importantly, a previous report described varying in vivo reactivity of lysine depending on its position in the albumin sequence and, thus, its neighboring amino acids^46^. Further, investigation of short-chain peptide model systems showed that AP formation is considerably influenced by the immediate chemical environment, hence, adjacent amino acids side chains in the peptide sequence^26,33^, which overall indicates that we may also have identified reactivity-sequence interrelation for peptide structures. We visualized the median percentage of each amino acid in APs to explore the effect of amino acid composition and microheterogeneity over all four casein proteins (Fig. 3c). Most amino acids showed a wide distribution of the values, again demonstrating that the type of amino acids that contribute to AP forming peptides can vary based on their immediate chemical environment. This presents a promising starting point to explore for sequence-specific glycation.

**Figure 4:**
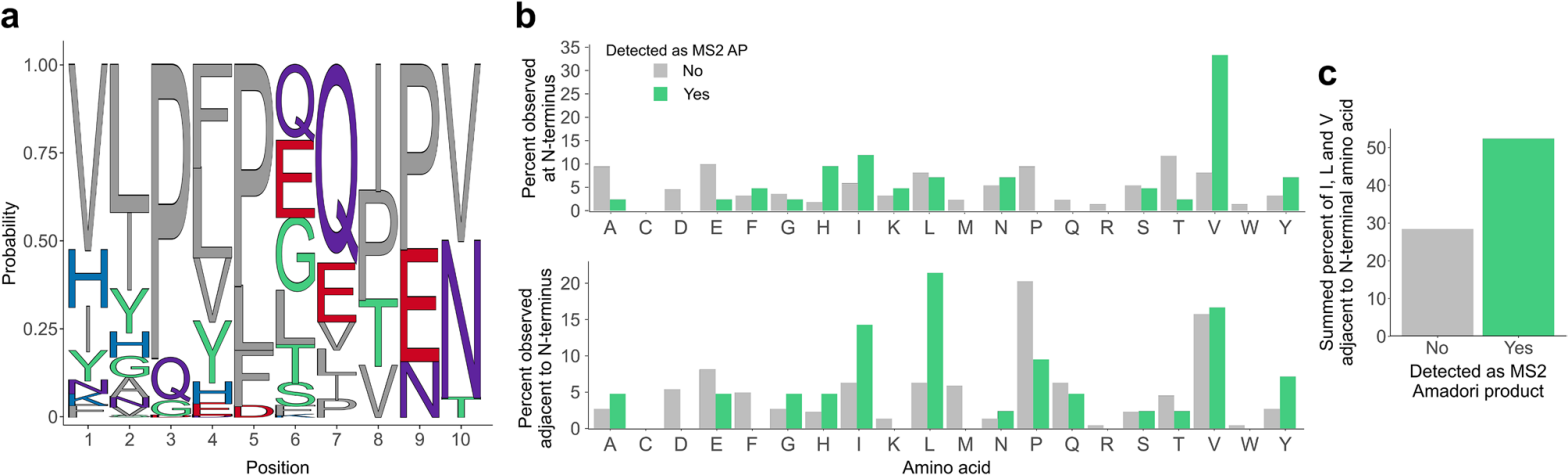
Analysis of the amino acid location relative to the glycation site reveals sequence-resolved changes in peptide reaction behavior. **(a)** Sequence logo representation of the first ten amino acid sequence positions of peptide APs. Amino acids with increased relative abundance in glycated peptides are illustrated (relative abundance_glycated_ – relative abundance_non-glycated_ > 0). (**b)** Comparison of amino acids at the N-terminal position and adjacent to the peptide N-terminus. Bars show the percentage of (glycated) peptides that contain a given amino acid at the first (top) and second position (bottom) of the amino acid sequence (detected as AP: green bars; not detected as AP: gray bars). (**c)** Summed percentage of peptides that contained isoleucine, leucine and valine adjacent to the N-terminal amino acid.

To dive into this intriguing facet of peptide glycation, we examined the location of amino acids relative to the reactive peptide N-termini . This was based on De Kok’s hypothesis that the side chain carboxylic group of Glu catalyzes glycation of primary amino groups^33^, and on the suggestion of Lhiang Zhili and co-workers that Leu and isoleucine (Ile) promote AP formation^26^. As short-chain peptides were investigated in these studies, they describe the influence of directly neighboring amino acid side chains on N-terminal peptide glycation. Hence, we reasoned that our dataset could allow to explore the impact of both the N-terminal amino acid and the adjacent amino acid side chain across a large number of highly diverse peptide species. To detect amino acid overrepresentation at the mentioned positions, we generated sequence logos by comparing sequences between peptide APs and non-glycated peptides (Fig. 4a and Supplementary Fig. 7a). Here, amino acids enriched at certain positions in AP forming peptides are illustrated (relative abundance_glycated_ − relative abundance_non-glycated_ > 0). This analysis indicated preference for valine (Val), Ile and Leu at the first two positions of the amino acid sequence for glycated peptides (Fig. 4a). Indeed, the percentage of N-terminal Val was considerably increased for AP forming peptides compared to peptides, for which the corresponding AP could not be identified (Fig. 4b, top)*.* An illustration of the absolute amino acid abundance can be found in Supplementary Fig. 7b. Interestingly, we further found substantially higher relative frequencies for Ile, Leu and Val next to the N-terminus in peptides also observed as an AP (Fig. 4b, bottom), echoing the result from the sequence logo. To account for preferred glycation of peptides with Ile, Leu or Val at the second sequence position, their summed relative frequency depending on AP detectability is shown in Fig. 4c. This result complies with previous findings that hypothesized that Ile and Leu promote N-terminal glycation based on pronounced hydrophobicity^51^ (Ile 1.80; Leu 1.70) and polarizability^52,53^ (Ile 91.21; Leu 91.60). Val has comparable physicochemical properties (hydrophobicity 1.22, polarizability 76.09) to Ile and Leu and was previously shown to exert *a* similar effect on the reactivity of the peptide N-terminus^26^*.* Furthermore, the percentage of aspartic acid (Asp), methionine (Met), phenylalanine (Phe) and Pro next to the N-terminus was considerably decreased in glycated peptides (Fig. 4b, top). We also observed that glycation of peptides was disfavored for Pro at the first two sequence positions (Fig. 4b and Supplementary Fig. 7a). A recent study on Pro containing dipeptides (Gly-Pro, Pro-Gly) suggested that its secondary amine may hinder Schiff base formation^54^. Nevertheless, we found that proline was frequently observed at the third and fifth sequence position of glycated peptides (Fig. 4a), thus raising the possibility of its involvement in increased peptide reactivity towards early glycation. This is supported by an increased relative abundance of proline at the same locations relative to the glycation site in AP forming peptides compared to peptides, for which the corresponding AP was not detected (Supplementary Fig. 7c and d).

### Capturing relevant sequence patterns in peptide glycation

**Figure 5:**
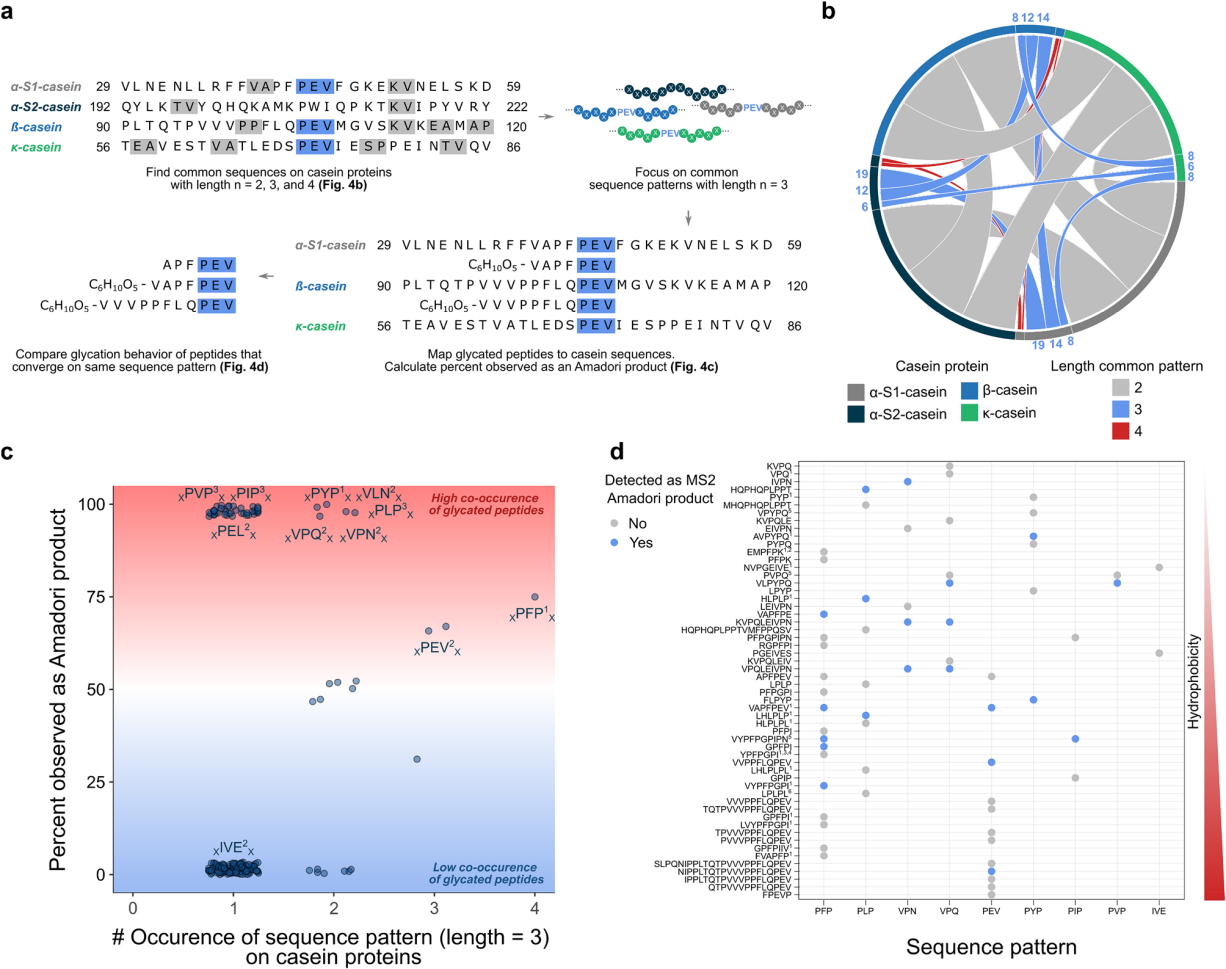
Mapping sequence patterns to protein and peptide domains exemplifies their role in peptide glycation. **(a)** Schematic illustration of sequence crosstalk decoding: Effect of sequence similarity on protein and peptide glycation based on presence of short-chain amino acid patterns. **(b)** A chord diagram representation of overlaying sequence patterns between casein proteins. The number of common di-, tri-, and tetra-sequence patterns was computed between each casein protein sequence. The size of the connections between the proteins (arcs) is relative to the number of common sequence patterns. **(c)** A scatterplot showing the percentage of sequence patterns with three amino acids in casein proteins covered by APs. Suspended numbers indicate sequence pattern dissimilarities as given in Supplementary Table 2. **(d)** Mapping sequence patterns to peptide domains. Peptides detected as an AP are colored in blue. Peptides that were not observed as the corresponding AP are shown in gray. Peptides are ordered by hydrophobicity according to their chromatographic retention time. The numbers denote established bioactive peptides (1 antihypertensive, 2 antimicrobial, 3 opioid, 4 immunomodulatory, 5 antioxidant, 6 DPP-IV-inhibitory).

Large-scale peptide derived AP analysis enables us to identify potentially relevant glycation-patterns, and our dataset can provide an initial glimpse into this intriguing aspect of glycation. While others have explored the influence of the amino acid sequence based on di- and tripeptide glucose model systems^26,33^, we can now comment on trends across 264 peptides originating from four proteins. In enzymatic glycosylation the importance of the N-X-S/T sequon and the negative effect of Pro in X position has been shown, which may result from conformational changes^55^. However, relevant structural motifs in peptide glycation have not been identified. In this detailed analysis, we identified small regions of identical subsequences in casein proteins and, thus, the thereof arising peptides (length = 2, 3, and 4) using the amino acid one letter code (Fig. 5a, top). We mapped (non-) glycated peptides onto proteins (Fig. 5a, bottom right part) and across each other (Fig. 5a, bottom left part). This provided information on the degree of co-occurrence for sequence patterns on glycated peptides with a different overall amino acid sequence. This protocol allows to detect relevant glycation patterns that are anticipated to be important factors in determining the preference for early peptide glycation. Analysis of common sequence patterns in casein proteins revealed several sequence overlays. Figure 5b provides information on the degree of sequence similarity, from which it is evident that the number of shared sequences varied for different pattern lengths and pairs of proteins. No shared tetra-sequences were found for κ-casein. To explore sequence patterns of maximum length, tri-patterns were chosen for further investigation. Figure 5c captures the total frequency of tri-sequence patterns in the casein protein sequences and which percentage of these differentially located subsequences was covered by peptide APs. This analysis allows to identify how different patterns contribute to glycation of peptides with shared subsequences but different overall amino acid composition as they originate from different casein protein regions. Of the three P-E-V sequons in casein proteins (P_44_–V_46_ of α-S1-casein and P_105_–V_107_ of ß-casein, Fig. 3a; P_171_–V_173_ of κ-casein, Supplementary Fig. 5), two appear as subsequences in peptide APs, as well as the alternated V-E-P (V_131_–P_133_ of ß-casein, Fig. 3a). Several interesting cases where substructures highly similar to P-E-V contribute to AP formation are highlighted (P-E-L, V-L-N, V-P-N, and V-P-Q; Fig. 5c). These subsequences all share amino acids with a low dissimilarity score (*D*), which is based on 134 categories of activity and structure^56^, and are partially rearranged (Supplementary Table 2). For example, P-E-V and P-E-L only differ by a single amino acid with strong physicochemical similarity (*D*(Val, Leu) = 9), whereas in case of P-E-V and V-P-Q (*D*(Glu, Gln) = 14) also the sequence order was changed. By comparison, peptides that contain I-V-E, which shows more pronounced sequence variations (*D*(Pro, Ile) = 24 and sequence rearrangement), do not participate in AP formation. All of these patterns, which show strong contribution to AP formation, either comprise Glu (carboxylic group), glutamine (acid amide group) or asparagine (acid amide group). A catalytic effect of the carboxylic group on AP formation was previously hypothesized^33^, which resembles the here found promoting effect of Glu-containing sequence patterns on the early MR. P-Y-P and P-F-P contain amino acids with comparable properties. The substructures P-I-P, P-L-P and P-V-P feature pronounced physicochemical similarities as well^56^. All these patterns showed a high co-occurrence on peptide APs relative to their total abundance (displayed as percent in Fig. 5c), which indicates their contribution to glycation independent of the overall peptide composition, thus their origin in the source protein . In contrast, P-N-P (P_198_–V_200_ of α-S1-casein) did not contribute to an AP (Fig. 3a) and shows pronounced differences in its amino acid characteristics.

A peptide-sequence pattern plot in Fig. 5d maps relevant sequence patterns to different (glyco-) peptides for which they could be observed. These peptides vary in their overall composition and peptide length. The map indicates, which patterns contribute to glycation on a peptide-level, and other peptide properties that considerably affect their reaction behavior. This analysis reveals several interesting trends, such as pronounced discrepancies in glycation of peptides with the same pattern and, perhaps most striking differences in AP formation of strongly related peptides. Small variations in the peptide amino acid sequence may cause (VVPPFLQPEV vs. VVVPPFLQPEV; YPFPGPI vs. VYPFPGPI) or not cause (VAPFPE vs. VAPFPEV; VYPFPGPI vs. VYPFPGPIN) differentiated behavior in the early MR. General correlation was not observed between AP formation and peptide physicochemical properties, expressed as hydrophobicity according to their retention time.

### System-wide analysis of bioactive and sensory-active peptide glycation enabled by in silico sequence mapping

**Figure 6:**
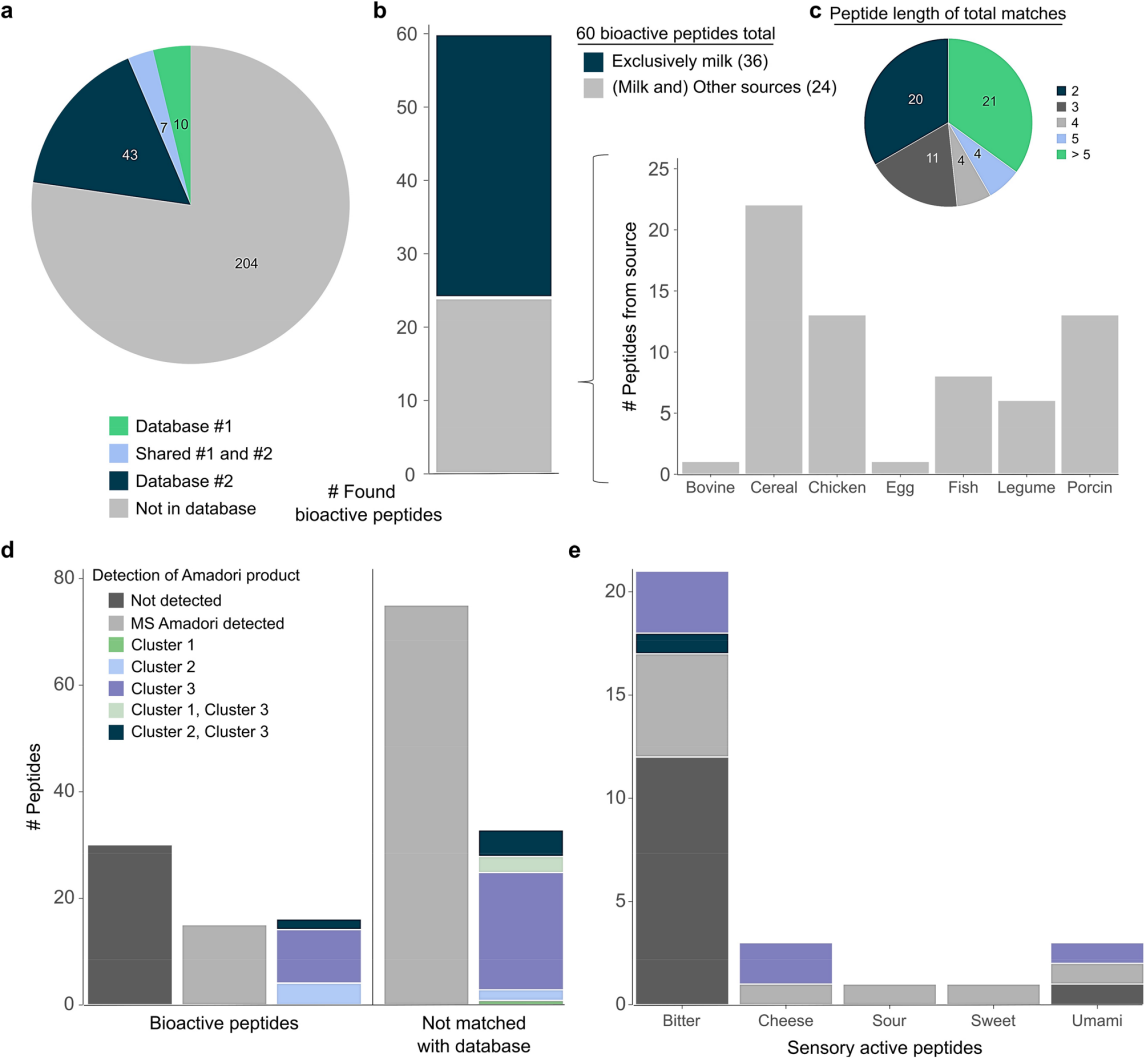
Database search uncovers overlap with miscellaneous peptide sources. **(a)** Identification of 60 peptides reported as bioactive peptides in databases for milk and other foods. (**b)** Classification of the assigned bioactive peptides according to different sources. Of these bioactive peptides, 36 are exclusively described as bioactive milk peptides (dark blue), and 24 are known bioactive peptides in other sources (gray). A bar plot represents the number of times peptides were matched with other sources than milk. Note, peptides that were found in multiple sources were counted for each source, individually. (**c)** Distribution of peptide lengths of those peptides which could be assigned to an established bioactivity. (**d)** The bar chart displays the number of bioactive peptides (left), and the number of peptides not matched with a database (right). Peptides were grouped into those, for which no AP (dark gray), an AP solely on precursor mass ± 10 ppm (light gray) or an AP also confirmed by MS/MS fragmentation pattern could be detected. APs with MS/MS fragmentation certainty were further classified into Cluster 1, Cluster 2, Cluster 3, Cluster 1 and 3 or Cluster 2 and 3 (see Fig. 2). (**e)** The bar graph depicts AP formation for 25 peptides with different sensory activities. If multiple sensory activities were reported for a peptide, it was counted more than once for this calculation.

The complexity of glycation represents a great challenge for the identification of glycation patterns that are associated with the gain or loss of bioactivity and glycation induced changes in sensory attributes. A combination of bioinformatics and database search enabled to study the established sensory and bioactivities of peptides in our dataset and to evaluate their behavior in glycation. We matched 60 peptides (Supplementary Table 3) with reported bioactivities (Fig. 6a), which were included in databases compiled from literature^57,58^. While 36 peptides were exclusively found in milk, 24 peptides appeared in a variety of other food sources as well (Fig. 6b). We also found that these peptides of diverse chain lengths (Fig. 6c) cover various bioactivity categories (Supplementary Fig. 8), suggesting that tryptone models may facilitate inter-disciplinary investigation of peptide glycation. In our study, for 62% of the bioactive peptides the corresponding AP was detected (33% confirmed by MS/MS tandem experiment; Fig. 6d). Hence, approximately 34% of the 47 peptides, for which the AP was identified via MS2 fragmentation, were previously reported to be bioactive. Furthermore, we identified 25 peptides with sensory attributes^59^. Figure 6e illustrates the prevalence of AP detection for sensory-active peptides and the level of AP identification (MS and MS2). Approximately 24% of all sensory-active peptides and 19% of the bitter peptides were observed as the corresponding AP on MS2 level. As noted by Shiyuan Dong and co-workers^32^, bitterness of MRPs was decreased compared to original casein peptides, and further reduced with heating time and glucose concentration. Xiaohong Lan et al. previously published that bitter soybean peptides below 1000 Da decreased 28.49% after reaction with xylose at 120 °C^60^. In contrast to the reported experiment, digesting casein with trypsin would produce large peptides, through protein cleavage after arginine and lysine, which would lead to the loss of highly interesting bioactive peptides (Supplementary Fig. 9) and would not allow comprehensive investigation of their reaction behavior (Supplementary Fig. 10 and Supplementary Fig. 11). For example, using a tryptic casein digest, the analysis of the opioid peptides, e.g. YPFPGPI and YPVEPF, for which N-glycation is known to have major consequences on the bioactivity of the parent peptide^61,62^, would not be possible. By comparison, in our experiment the large and diverse peptide spectrum of tryptone enabled us to widely predict bioactive peptide glycation.

### Convergence of tryptone peptides and peptides with established activities into common sequences

**Figure 7:**
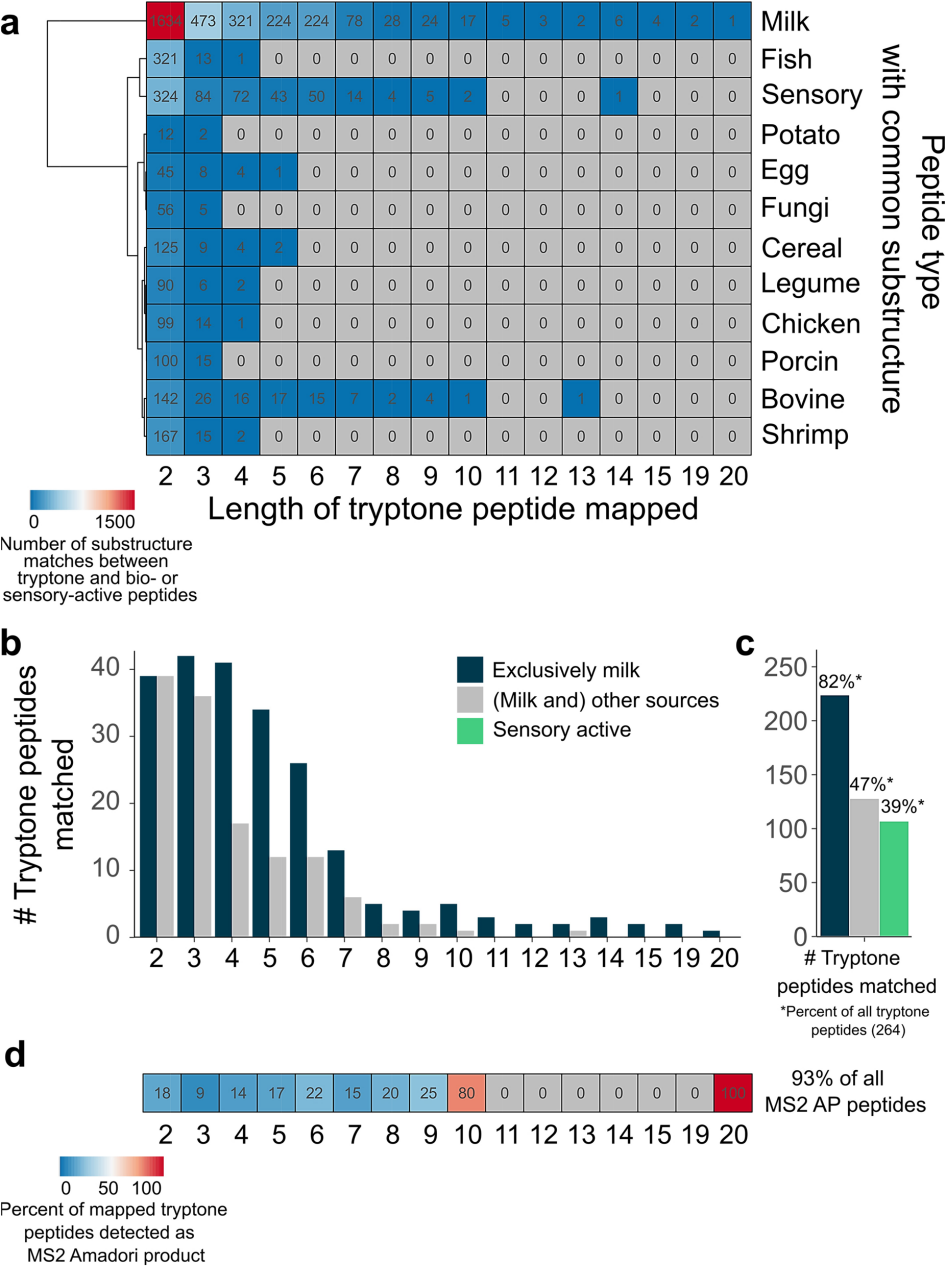
Mapping of tryptone peptides to established bioactive peptides indicates a wide scope of application. **(a)** Tryptone peptides were mapped on sensory-active peptides and bioactive peptides from 11 sources by substring matching to explore similarity in their amino acid sequence. A sequence co-occurrence heatmap indicates the number of sequence overlaps between tryptone peptides of a certain length and bio-/sensory-active peptides, with colors indicating the number of matches. Gray denotes zero sequence overlays. (**b)** Absolute number of tryptone peptides successfully mapped to bioactive peptides from milk (dark blue) or other sources (gray) depending on the peptide length. (**c)** Total number of tryptone peptides identified as subsequence of bioactive peptides in milk (dark blue, 82% of all tryptone peptides) or in other sources (gray, 47%) and of sensory-active peptides (green, 39%). (**d)** Heatmap illustrating the percentage of the matched tryptone peptides that were detected as AP. Approximately 95% of all AP forming peptides were identified as a subsequence of bioactive peptides.

Given that similarities in the amino acid sequence and sequence patterns may determine the reaction behavior of peptides, we reasoned our approach could provide insight into peptide reactivity from a systems level. To test this hypothesis, we searched peptides from our dataset as a pattern of bioactive and sensory-active peptide sequences reported in databases by substring matching^57–59^. First, we examined the number of matches observed. In total, tryptone peptides were successfully mapped to 1172 unique bioactive peptide species (Supplementary Fig. 12). Even though caseins are major proteins in milk, a considerable number of common sequences between tryptone peptides and a plethora of peptides from other sources was found (Fig. 7a). Overall, we achieved 3046 sequence overlays with 675 bioactive milk peptides, 1350 overlays with 510 peptides from other sources, and 599 overlays with 174 sensory-active peptides (Supplementary Fig. 13). Importantly, not only small sequence commonalities were found as indicated by the length of the tryptone peptides mapped (Fig. 7a). Tryptone amino acid sequences, up to a length of thirteen amino acids, occurred on bioactive peptides from entirely different origins. Even larger sequences (n = 14) co-occurred in peptides with established sensory activity. The heatmap (Fig. 7a) visualizes classes of peptides delineated by the number of overlays per peptide length, showing increased sequence similarities for sensory-active peptides and fish. Or, in case of potato peptides we found a lower proportion of common di- and tri-sequences compared to other sources and no matches for larger peptides (n > 3) were observed. We note that calculating co-occurring sequences is straightforward and may provide information about glycation susceptibility of specific peptide classes from various protein origins. Supplementary Fig. 14 demonstrates that a broad variety of bioactivities is covered by the database peptides, to which tryptone peptides were successfully mapped. Other trends arise, such as the presence of a relatively high abundance of antihypertensive peptides. Note, this is expected because of the inordinate number of antihypertensive peptides in the databases used. The same number of dipeptides was successfully mapped to bioactive peptides independent of the source (Fig. 7b), and the number of tripeptides found as a subsequence was comparable as well. For larger tryptone peptides, approximately half of the peptides matched for milk were found to overlay with peptides, which were (also) found in other food sources. A striking feature of this analysis are the percentages of peptides in our dataset found as a subsequence (Fig. 7c). Considering the 264 peptides that could be mapped to a domain, 82% existed in bioactive peptides exclusively from milk and 47% in those from (milk and) other sources. Furthermore, 39% of tryptone peptides were successfully mapped to sensory-active peptides (Fig. 7c). Figure 7d depicts the percentage of matched tryptone peptides, for which the corresponding AP was identified. Up to 22% of the shorter substring peptides (n ≤ 6 amino acids) were detected as an AP by tandem MS experiments, while the majority of larger peptides appeared to be glycated. In total, 93% of the peptides detected as an AP are substructures of bioactive peptides. This represents a considerably larger proportion compared to other peptide groups, e.g. sensory-active peptides (Fig. 7d).

## Discussion

In all, here we present a straightforward approach to refine evaluation of peptide derived APs by using the power of high-resolution MS in combination with multivariate statistics and bioinformatics to access large-scale information about peptide reactivity in the MR and the influence of both reaction time and sugar concentration. Investigation of glucose-tryptone model systems enabled the most in-depth profiling of peptide APs to date. By comparison with a in silico tryptic casein-digest, we demonstrated considerable advantages of tryptone models, such as a notably larger coverage of (bioactive) peptides from various food sources. This strategy is amenable to virtually any type of MR model system or reactivity study with known reaction intermediates. Finally, the reaction behavior of 264 casein derived peptides was characterized by AP analysis from a single type of model system, which demonstrates that new models must be developed to unravel the glycation reaction network in its full complexity. Clearly, large-scale studies are needed to explore peptide glycation and its importance particularly in food but also biological systems and, thus, health.

A caveat of practically any MS-based experiment is that detectability can be affected by the type of ionization, analyte concentration as well as sample complexity. Thus, there may be a bias toward specific peptides and APs to consider in this dataset. Furthermore, Fig. 1c shows a high frequency of tryptone peptides from certain protein regions , which may arise from its production process and above-mentioned detectability issues. Our data interpretation, however, reflects on ubiquitous observations in the overall dataset, and not on specific peptide species . Despite this, we achieve in-depth characterization of a large reservoir of peptides and provide thorough information on peptide properties influencing AP formation. We relied on normalized AP intensity profiles for reaction behavior investigation, meaning there are limitations in the stability of this early reaction intermediate to consider. Greifenhagen et al. has noted pronounced susceptibility of the N-terminally acetylated Amadori peptide Ac-Ala-Lys-Ala-Ser-Ala-Ser-Phe-Leu-NH_2_ toward oxidative degradation in aqueous model systems^63^. Loss of the Amadori compound of the endogenous opioid pentapeptide leucine-enkephaline (Tyr-Gly-Gly-Phe-Leu) was also noticed by Jakas and Horvat^64^, however, markedly slower degradation behavior was observed in this study. Different reaction conditions, such as concentration of catalytically active phosphate buffer and temperature, tend to affect AP stability, but also the amino acid sequence of the peptides. Even with this, all peptide APs detected in this dataset remained above the limit of detection at all time points.

While other studies have investigated glycation using a limited number of highly specific synthetic peptides, we could simultaneously study the reaction behavior of a large pool of casein peptides. We can also see similar trends to previous studies, such as the influence of both reaction time and sugar concentration^65,66^. In contrast, we can provide detailed information on how APs derived from specific peptide sequences are affected. We show that upon reaction time the bulk of peptides differentiated into three clusters, reaching maximum AP intensity at different time points and, thus that AP formation rates likely depend on the peptide structure. Peptides forming APs that peaked at early reaction time points and low glucose concentrations may represent more reactive precursors in glycation. A consecutive decrease in relative AP peak intensity may be attributed to further rearrangement and oxidative cleave reactions yielding heterogeneous AGEs^67^. Further, we show that there is no general correlation between peptide length and reactivity. More pronounced susceptibility of dipeptides compared to tripeptides toward glycation was seen in early studies of glycine (Gly) peptide model systems (GlyGly > GlyGlyGly) and in more miscellaneous synthetic peptide studies^26,33,34^. Similarly, we found higher proportions of glycated dipeptides than tripeptides. Although our observations are in congruence with previous studies, we are the first to investigate the impact of peptide length at this scale providing a new perspective on its influence on reactivity. We also show that there is not a general correlation between amino acid content and susceptibility towards the initial phase of glycation reactions. This suggests a strong contribution of other factors such as the amino acid sequence, thus, the amino acid microenvironment.

In peptide glycation, reaction behavior has been proposed to be driven by the amino acid sequence. An important role of the amino acid adjacent to the N-terminus has been suggested based on short-chain peptide model systems^26,33,34^. Here, we noticed strong preference of glycation for valine-starting peptides and noted more pronounced AP formation of peptides, which contain Ile, Leu and Val positioned next to the N-terminal amino acid. Further, we observed prevalence of Met, Phe, and especially Pro at the second sequence position in peptides, for which the corresponding AP could not be identified, which indicates that steric hindrance or conformational changes may prevent N-terminal glycation. Congruent observations were made for the N-X-S/T sequon, where Pro in the X position causes pronounced changes in conformation and, thus prevention of enzymatic glycosylation^55^. We show that neighboring Glu, for example, may not always exert a catalytic effect on N-terminal glycation as a result of its carboxylic side chain. Interestingly, APs from peptides containing Asp adjacent to the N-terminus were not observed, even though its structure is closely related to Glu. Depending on the amino acid sequence and the peptide N-terminus, differentiated effects of the neighboring amino acid may be observed^33^, and here, we can cover a broad range of diverse peptide properties. Such thorough and integrated characterization of peptide APs depending on the reaction conditions is necessary for a complete understanding of peptide glycation and its impact on food and biological systems. We further found two location sites near the N-terminus with increased relative abundance of Pro in AP forming peptides, namely the third and fifth position of the amino acid sequence.

We identified peptide collectives particularly prone to early glycation reactions by mapping APs to casein sequences and across each other. Furthermore, we leverage these reactive peptide species to provide information on potentially important tri-sequence patterns and propose that glycation patterns among many other factors promote peptide glycation, which is indicated by strong connectivity in glycation susceptibility and presence of specific sequence patterns. Even though N-terminal proline may inhibit Schiff base formation^54^, here, we established several proline-rich sequence patterns, which considerably triggered AP formation. Furthermore, Glu containing sequence patterns, such as P-E-V, may exert a catalytic effect towards early MR^33^. Depending on whether glycation is desired or not, peptides may be chosen, accordingly (discussed below). Overall, we provide a comprehensive set of molecular checkpoints for peptide reactivity estimation towards glycation.

Finally, we established system-wide applicability of tryptone model systems by mapping tryptone peptides to a plethora of bioactive and sensory-active peptides from various food sources. Depending on whether glycation of peptides is desired or not, we suggest that the amino acid sequences may be chosen, accordingly. For development of functional foods, health benefits must be preserved, thus for most bioactive peptides AP formation needs to be obviated. For example, for opioid peptides, the requirement of free N-terminal tyrosine was demonstrated and the loss of antihypertensive properties of casein peptides as a result of glycation was revealed in various model systems^29,30,68^*.* Conversely, if increased antioxidant potency^26,69^ must be achieved, we suggest that the peptide species may be capitalized that are more prone to AP formation. The increased antioxidative properties of MRPs compared to their corresponding casein peptides has previously been determined^27,28,30^, and consequently targeted peptide glycation may enable to enhance the health benefits of peptides. Superior antioxidative properties have been established for MRPs derived from small peptides compared to larger peptide species^69^, which makes tryptone particularly suitable for identification of potential peptide candidates. In total, we observed APs from 47 amino compounds. For 34% of them, bioactivity was previously established and 93% were identified as a substructure of bioactive peptides, which suggests that bioactive peptides are particularly prone to glycation. For sensory-active peptides, others have observed reduced bitterness of MRPs compared to heated casein peptides alone, while antioxidative properties where increased^26^. Similar findings were reported for peptides from other sources^60,70,71^. Even more benefits for food production can thus be provided by choosing appropriate peptide candidates, such as enhanced sensory attributes of foods. To assess desired flavor improvement^71,72^, we reasoned that selection of peptides susceptible to early glycation may be promising. As bitter peptides cannot be employed above their bitter flavor threshold^72^, increased bioactivity accompanied concurrently by decreased bitterness may be desirable for the production of functional foods to improve health and enhance customer acceptance ^23^. Taken together, our dataset allows to select suitable peptide candidates, given (1) a checklist for estimation of their reaction behavior in early glycation reactions according to the amino acid at the N-terminus, the adjacent sequence position and presence of relevant sequence patterns, and (2) screening for established sensory attributes and bioactivity.

Future studies are required to investigate a wider range of peptides from different proteins and, thus a broader variety of amino acid sequences to gain more global information on the relevance of amino acid composition and sequence patterns. Model systems prepared from highly specific synthetic peptides have provided valuable findings in previous studies, which suggests that targeted investigation of peptides, in particular with potentially relevant sequence patterns, may be promising for identification of peptides especially prone to peptide glycation. These strategies also present an opportunity for determination of peptides less susceptible toward glycation reactions. Investigation of changes in sensory attributes and bioactivity as a result of glycation will be a worthwhile endeavor in future experiments to gain the necessary refined information for systematic use of specific peptides and their glycation products as functional food ingredients.

## Methods

### Preparation of model systems

d-( +)-glucose (> 99.5%) and tryptone were purchased from Sigma-Aldrich (Steinheim, Germany). Tryptone (6% (w/v)) was mixed with glucose at different concentrations (0.015 M, 0.03 M, 0.15 M, 0.3 M) in MilliQ-purified water from a Milli-Q Integral Water Purification System (18.2 MΩ, Billerica, MA, USA). Aqueous tryptone solutions (6% (w/v)) were prepared as control samples. Model systems were heated in closed glass vials for two, four, six and ten hours at 95 °C according to the protocol recently described^42^. Control samples were heated for 10 h, analogously. Sample preparation was performed in triplicate (n = 3). Model systems were stored at −20 °C.

### LC–MS/MS

Model systems were diluted 1:6 (v/v) with an aqueous solution containing 2% acetonitrile (LC-MS grade, Merck, Darmstadt, Germany) prior to LC-MS/MS analysis. Samples were analyzed by UHPLC (Acquity, Waters, Milford, MA, USA) coupled to a quadrupole time-of-flight (Q*q*TOF) mass spectrometer (MS) (maXis, Bruker Daltonics, Bremen, Germany). For reversed phase (RP) chromatography an ACQUITY UPLC BEH C18 column (100 × 2.1 mm, 1.7 µm, Waters, Milford, MA, USA) was used. The column temperature was maintained at 40 °C. RP separation was run in gradient mode. The RP eluent A was a composition of 0.1% (v/v) formic acid and RP eluent B was composed of acetonitrile with 0.1% (v/v) formic acid . Pre-equilibration time was set to 2.5 min. Initial conditions were set to 95% eluent A and 5% eluent B. This composition was maintained until 1.12 min. Eluent B was increased to 99.5% within 5.29 min, maintained to the end of the run. The gradient was completed after 10.01 min. Samples were injected via partial-loop-injection (5 µL). Mass spectra were acquired in positive electrospray ionization mode. Internal calibration was performed using a tuning mix solution (Agilent Technologies, Waldbronn, Germany) prior to each measurement. Parameters of the ESI source were: capillary voltage 4000 V, dry gas temperature 200 °C, nebulizer pressure 2 bar, and nitrogen flow rate 10 L/min. Mass spectra were recorded with an acquisition rate of 5 Hz within a mass range of *m/z* = 50–1500. For data-dependent MS/MS acquisition, the most abundant ion of a full MS scan was subjected to MS/MS after each precursor scan. The collision energy was set to 35 eV and to change dynamically and proportionally to the mass of the precursor molecule. Raw data were post-processed using GeneData Expressionist Refiner MS 13.0 (GeneData GmbH, Basel, Switzerland) applying chemical noise subtraction, intensity cutoff filter, calibration, chromatographic peak picking, and isotope clustering. Only features detected in all three replicates were retained in the matrix.

Chemical peptide structures were confirmed by peptide mapping in GeneData Expressionist Refiner MS 13.0 (GeneData GmbH, Basel, Switzerland) with an absolute *m/z* tolerance of 0.005 and 0.1 for the precursor and product ions, respectively, unspecific enzyme cleavages, a minimum peptide length of 1 AA, and no fixed or variable modifications. The fragmentation type was set to ESI CID/HCD. The peptide mapping was performed using a text file containing four AA sequences in FASTA format of bovine milk caseins including α-S1-, α-S2-, β-, and κ-casein. Top-down sequencing annotations for each of the four casein proteins were exported from Refiner MS module providing a list of the identified peptides along with their positions in the protein sequence they were successfully mapped to. Further processing was performed in R software (version 3.5.2). Amadori product precursor mass was calculated by a mass increase of 162.0528 Da. Amadori product precursor signals were computationally assigned by an algorithm within a mass tolerance of ± 10 ppm. Putatively assigned Amadori products with available MS2 spectra from our data were clustered according to their similarity in normalized intensity profiles using Pearson correlation. Product ion annotation was automatically performed in R software by in silico fragmentation^43,73^ and manually validated. Monoisotopic mass tolerance was set to ± 0.005 Da for product ions. To separate false positive assignments, we excluded signals with a poor fit of the MS/MS spectrum to the in silico predicted fragments and maximum intensity in the tryptone control samples heated for 10 h. GeneData Expressionist Refiner MS 13.0 (GeneData GmbH, Basel, Switzerland) peptide mapping activity provides a consolidated score, which describes the average fit for each peptide across all MS2 spectra available. Consolidated scores of all peptides, for which the corresponding Amadori product (MS2 level) could be verified, were computed. The minimum consolidated score per peptide length was chosen as a threshold for peptide identification.

### Statistical analysis

Pearson correlation coefficients were calculated in R software between intensity values for putatively assigned MS2 Amadori products (n ≥ 3, p ≤ 0.05). For this analysis, relative intensity values were used. Relative intensity values were calculated by normalizing intensity values to the maximum intensity value across all time points and for each Amadori product, respectively. Hierarchical clustering to provide the domain ordering was done using R software. Amadori product wise distances were calculated based on these correlations using the as.dist() function followed by hierarchical clustering using the hclust() function. To assess the importance of small sequence variations, pairwise two-sided t-tests were performed. Intensity values in model systems and control samples were compared. Significantly increased Amadori products (*p* ≤ 0.05) and relevant reaction conditions are reported in Supplementary Table 1. Multiple testing correction was performed using the Benjamini-Hochberg procedure.

### Sequence grouping

The computational analysis of sequence groups was performed with the peptide single letter code using R software. To identify peptides with common sequences the grepl() and match() base functions were applied. Based on derived sequence commonalities, we assigned all peptides to sequence groups (Supplementary Table 1). Amino acids were not assigned to sequence groups. Sequence groups, for which no Amadori product was detected, were excluded in Supplementary Table 1.

### Database search

Bioactive peptide database search was carried out using the Milk Bioactive Peptide Database (March 13, 2020)^57^ and the BioPepDB database (March 13, 2020)^58^. Sensory peptide database search was performed using the BIOPEP database (March 13, 2020)^59^. Database queries and substring search of tryptone peptides in bioactive peptides were conducted with the peptide sequence single letter code using R software. Hierarchical clustering to provide the bioactive peptide source ordering was done in R software.

## Supplementary Information

Supplementary Information.

Supplementary Table S1.

Supplementary Table S2.

Supplementary Table S3.

## Data Availability

The authors declare that the main data supporting the findings of this study are available within the article and its Supplementary Information files. Extra data are available from the corresponding authors upon request.
