# Supplementary material for: Molecular characterization of sequence-driven peptide glycation
Source: Sci Rep. 2021 Jun 24;11:13294. doi: 10.1038/s41598-021-92413-7 (PMC8225897; doi:10.1038/s41598-021-92413-7)
Supplement: Supplementary file 1 — Supplementary Information. [file 41598_2021_92413_MOESM1_ESM.pdf]

SUPPLEMENTARY INFORMATION

Molecular characterization of sequence-driven peptide glycation

Michelle T. Berger[1,2,*], Daniel Hemmler[1,2], Alesia Walker[2], Michael Rychlik[1], James W. Marshall[3], and Philippe Schmitt-Kopplin[1,2,*]

1. *Chair of Analytical Food Chemistry, Technical University Munich, Maximus-von-Imhof-Forum 2, 85354 Freising, Germany*
2. *Research Unit Analytical BioGeoChemistry (BGC), Helmholtz Zentrum München, Ingolstädter Landstrasse 1, 85764 Neuherberg, Germany*
3. *The Waltham Pet Science Institute, Mars Petcare UK, Waltham-on-the-Wolds, Leicestershire LE14 4RT, United Kingdom*

* .. Correspondence to Michelle Tamara Berger (michelle.berger@tum.de) and Philippe Schmitt-Kopplin (schmitt-kopplin@helmholtz-muenchen.de)

SUPPLEMENTARY TABLES AND FIGURES

Supplementary Table 1. Amino compound and Amadori product characteristics.

Supplementary Table 2. Dissimilarity of important glycation patterns (length = 3) from Figure 4.

Supplementary Table 3. Bioactive peptide identities from Figure 5.

Supplementary Figure 1. Tryptone peptides (n = 433 sites, upper protein sequences) provide substantially larger number of cleavage sites compared to a theoretical digest of casein proteins with trypsin (n = 79 sites, lower protein sequences).

Supplementary Figure 2. Tryptone promotes N-terminal diversity in arginine- and lysine-free peptides compared to a tryptic casein digest.

Supplementary Figure 3. *In silico* tryptic casein digest provides increased peptide lengths compared to tryptone peptides.

Supplementary Figure 4. Intensity profiling enables Amadori product classification by their formation behavior.

Supplementary Figure 5. Unraveling casein protein sequences prone to AP formation.

Supplementary Figure 6. Analyzing amino acid distribution displays their contribution to peptide glycation.

Supplementary Figure 7. Peptide sequence analysis reveals interesting trends for the location of amino acids relative to the glycation site.

Supplementary Figure 8. Database search reveals broad coverage of bioactivity categories by tryptone peptides.

Supplementary Figure 9. Sequence analysis of ß-casein$_{49-112}$ demonstrates omission of several bioactive tryptone peptides after *in silico* tryptic digestion.

Supplementary Figure 10. Bioactive peptides identified for a theoretical tryptic casein digest.

Supplementary Figure 11. Distribution of the peptide length uncovers poor coverage of bioactive short-chain peptides for a theoretical casein digest.

Supplementary Figure 12. Substring matching shows substantial sequence overlap of tryptone peptides and reported bioactive peptides.

Supplementary Figure 13. Mapping tryptone peptides to established bioactive peptides exposes considerable sequence commonalities with various sources.

Supplementary Figure 14. Peptides with miscellaneous bioactivities show common subsequences with tryptone peptides.

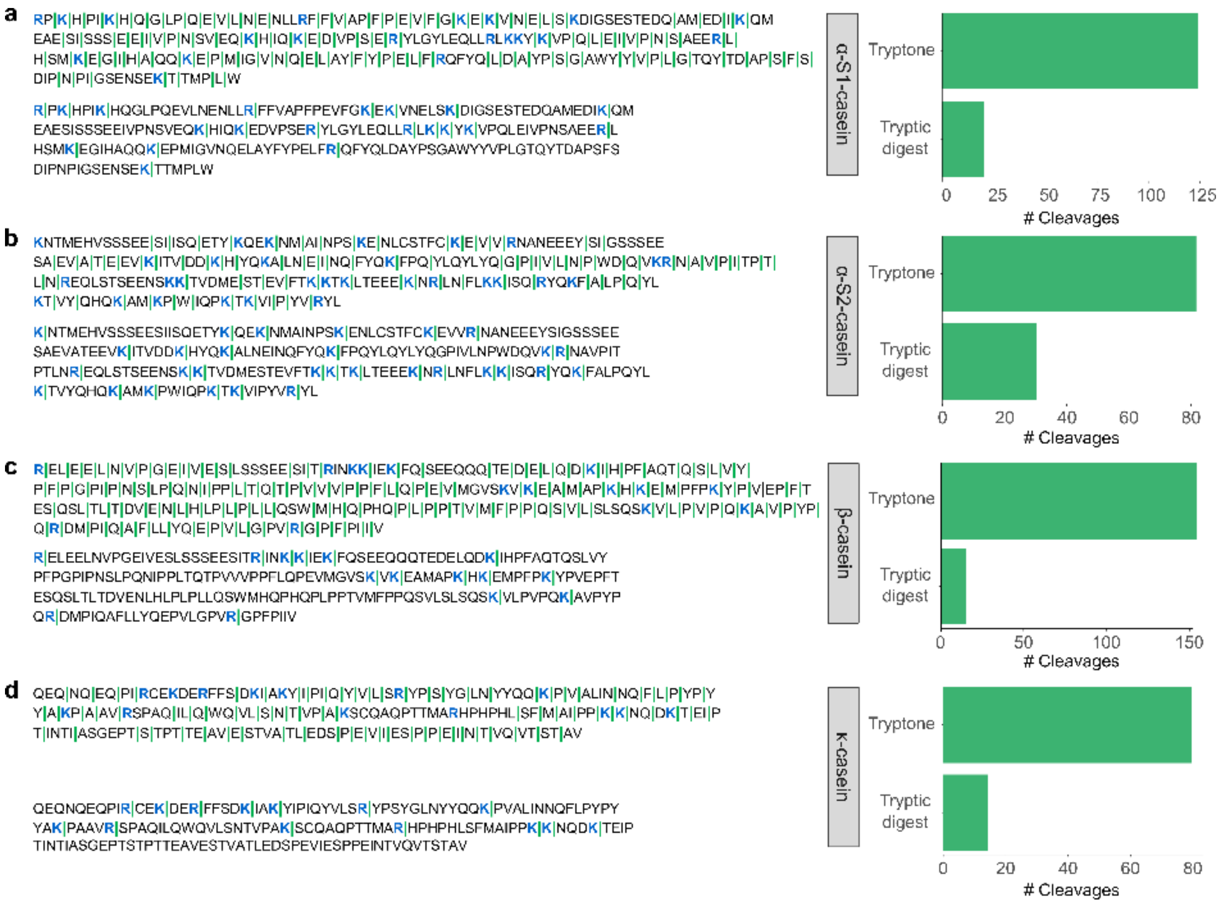

Supplementary Figure 1. Tryptone peptides (n = 433 sites, upper protein sequences) provide substantially larger number of cleavage sites compared to a theoretical digest of casein proteins with trypsin (n = 79 sites, lower protein sequences). Detected tryptone peptides were used to compute cleavage sites on casein proteins (upper protein sequences). Theoretical cleavage sites for a tryptic casein digests are highlighted in the lower protein sequences, respectively. Vertical bars (|) indicate cleavage positions, and lysine (K) and arginine (R) are highlighted in blue to provide an eye guide for amino acids that lead to a protein cleavage by trypsin. Bar plots provide the total number of cleavage sites per protein sequence in tryptone and in a theoretical tryptic digest.

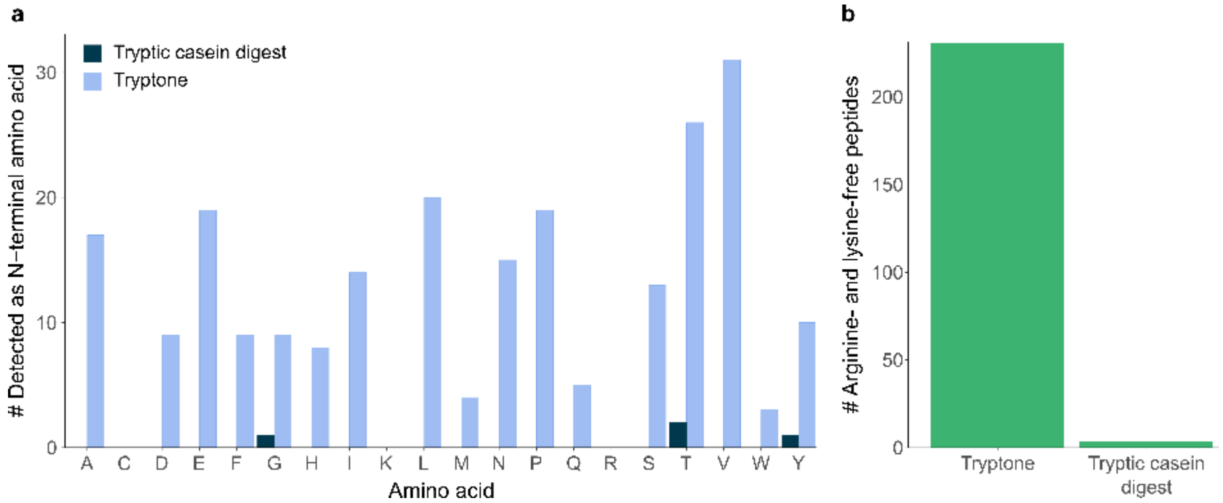

Supplementary Figure 2. Tryptone promotes N-terminal diversity in arginine- and lysine-free peptides compared to a tryptic casein digest. a) Bar graph shows N-terminal amino acids for arginine- and lysine-free peptides detected in tryptone (light blue) compared to arginine- and lysine-free peptides that can be expected after a tryptic digest of casein (dark blue). For the *in silico* digestion, α-S1-, α-S2-, β-, and κ-casein were considered. **b)** Total number of arginine- and lysine-free peptides for tryptone and a theoretical tryptic casein digest.

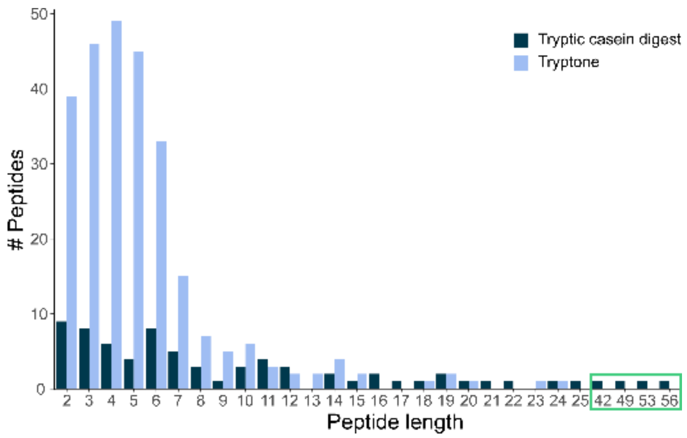

Supplementary Figure 3. *In silico* tryptic casein digest predicts increased peptide lengths compared to experimentally determined tryptone peptides. Bar chart depicts length of peptides detected in tryptone and after a theoretical casein digest with trypsin. For this calculation, α-S1-, α-S2-, β-, and κ-casein were considered. Note the markedly large peptides generated by using trypsin (highlighted by green box).

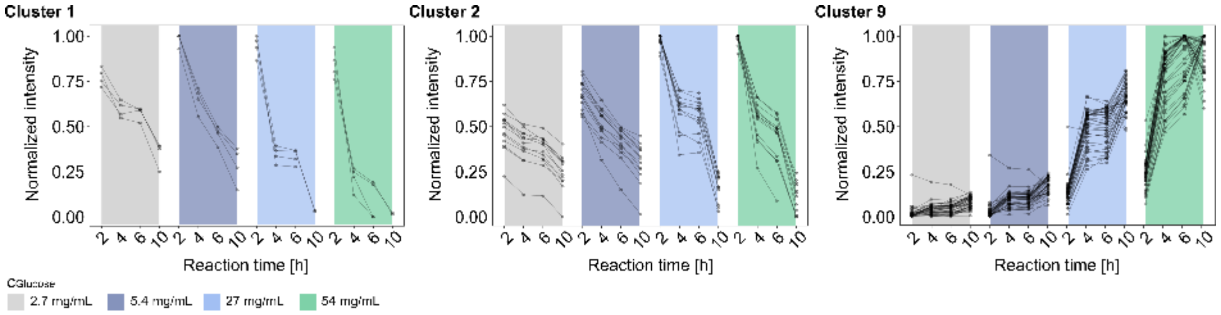

Supplementary Figure 4. Intensity profiling enables Amadori product classification by their formation behavior. Intensity profiles visualize time-resolved AP formation depending on the glucose concentration. Intensity values were normalized towards the greatest intensity value. Colors indicate different glucose concentrations.

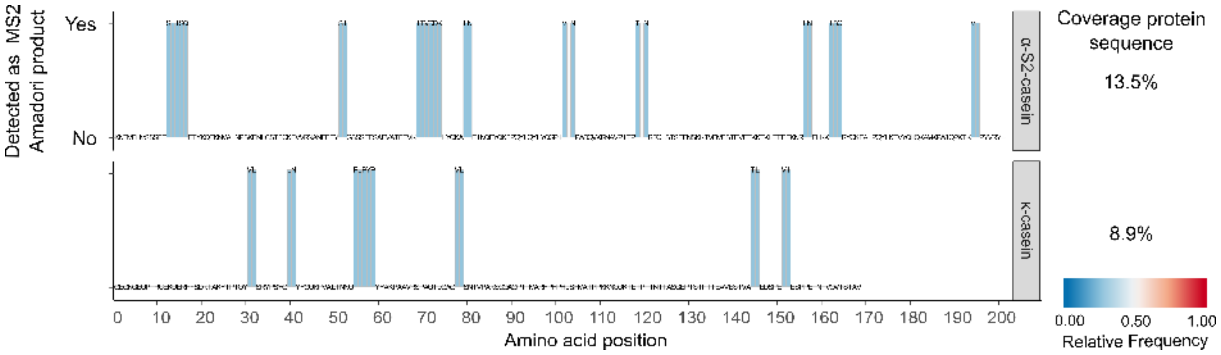

Supplementary Figure 5. Unraveling casein protein sequences prone to AP formation. α-S2- and κ-casein protein heatmaps represent the relative frequency of amino acid positions that were detected as an AP, indicating which protein regions contribute most to early MR and how the amino acid microenvironment influences peptide reaction behavior. Approximately 13.5% of α-S2- and 8.9% of κ-casein are detected as the corresponding AP.

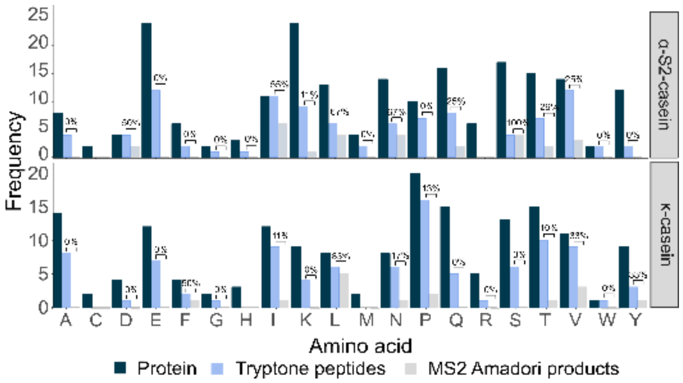

Supplementary Figure 6. Analyzing amino acid distribution displays their contribution to peptide glycation. Abundance of amino acids in casein proteins (dark blue), tryptone peptides (light blue) detected APs (gray). Embedded values indicate the percentage of peptides that could also be detected as an AP.

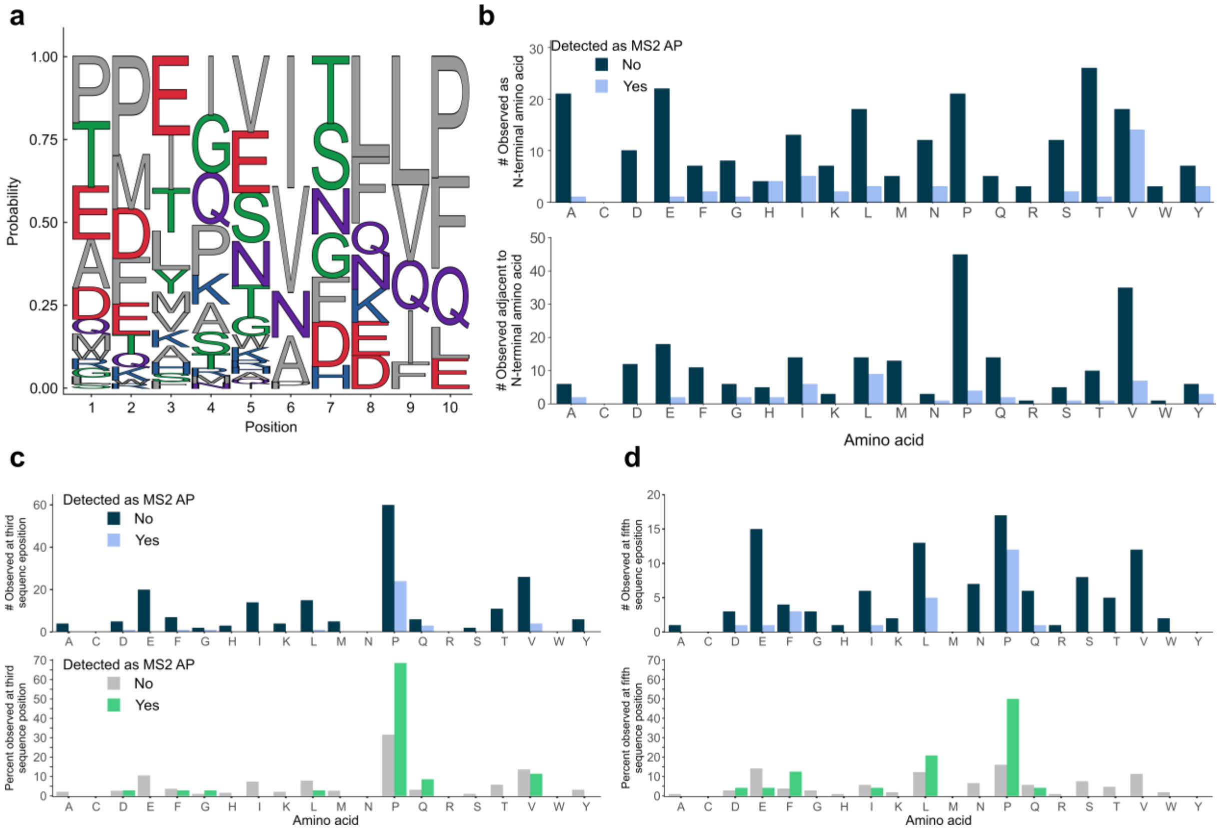

Supplementary Figure 7. Peptide sequence analysis reveals interesting trends for the location of amino acids relative to the glycation site. a) Sequence logo representation of the first ten amino acid sequence positions of non-glycated peptides. Amino acids with increased relative abundance in non-glycated peptides are illustrated (relative abundance$_{\text{non-glycated}}$ – relative abundance$_{\text{glycated}}$ > 0). **b)** Bars represent the absolute number of amino acids detected at the first (top) and second (bottom) sequence position in peptides, which were not observed as an AP (dark blue) or for which the corresponding AP could be detected (light blue). **c)** Bars show the absolute (top) and relative (bottom) frequency of (glycated) peptides that contain a given amino acid at the third position of the amino acid sequence (detected as AP: light blue (top) and green (bottom); not detected as an AP: dark blue (top) and gray (bottom)). **d)** Same illustration as shown in c) but for the fifth position of the peptide amino acid sequence.

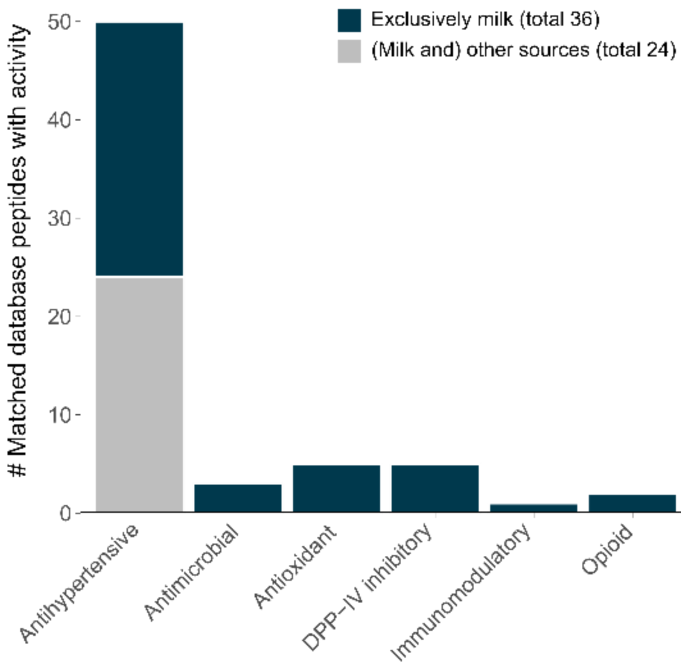

Supplementary Figure 8. Database search reveals broad coverage of bioactivity categories by tryptone peptides. Bars show the number of matched peptides exclusively found in milk (dark blue bars) and found in other sources (gray bars) and compare different bioactivity categories.

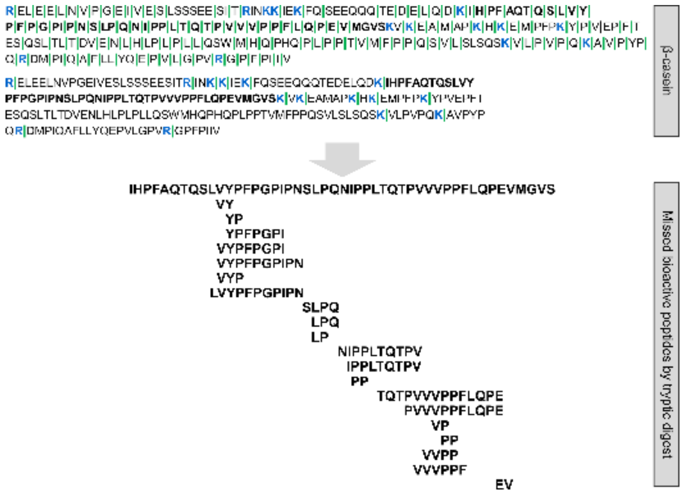

Supplementary Figure 9. Sequence analysis of ß-casein$_{49\text{-}112}$ demonstrates omission of several bioactive tryptone peptides after *in silico* tryptic digestion. Comparison of cleavage sites on ß-casein for tryptone peptides (top) and a theoretical digest with trypsin (bottom). Vertical bars (|) indicate cleavage positions. Lysine (K) and arginine (R) are highlighted in blue to indicate trypsin cleavage positions. Bold letters denote ß-casein$_{49\text{-}112}$ as produced by trypsin digestion and the corresponding tryptone peptides with established bioactivities.

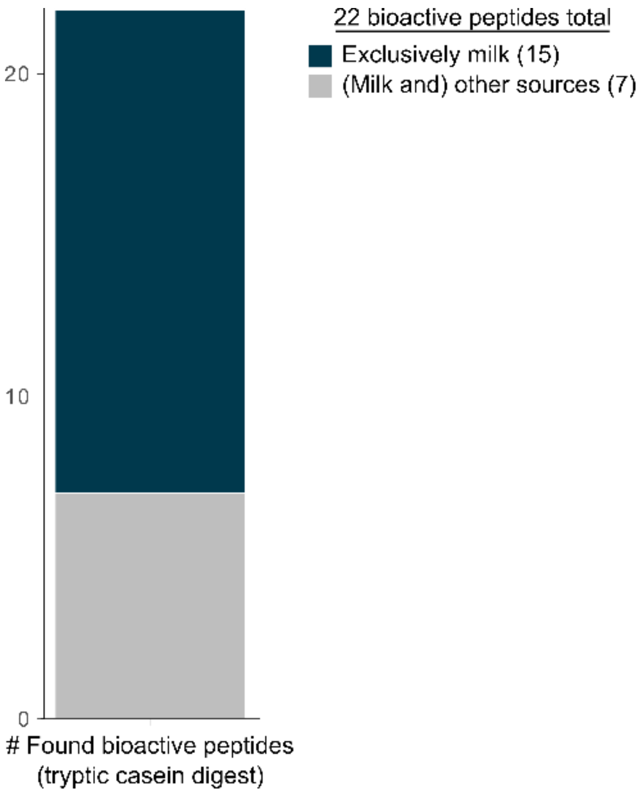

Supplementary Figure 10. Bioactive peptides identified for a theoretical tryptic casein digest. Of 22 peptides with established bioactivities, 15 are exclusively described as bioactive milk peptides (dark blue), and 7 are known bioactive peptides in other sources (gray). For this calculation, α-S1-, α-S2-, β-, and κ-casein were considered.

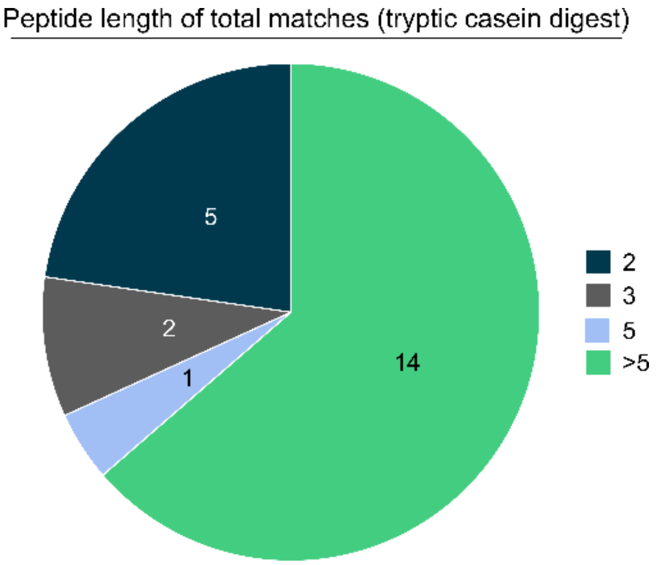

Supplementary Figure 11. Distribution of the peptide length uncovers poor coverage of bioactive short-chain peptides for a theoretical tryptic casein digest. Pie charts show the length of peptides for which a possible bioactivity could be found.

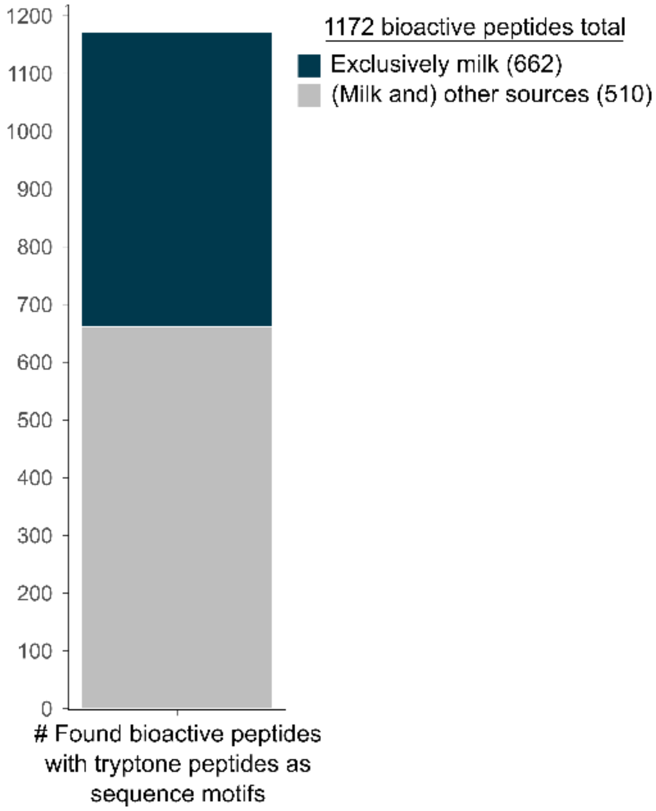

Supplementary Figure 12. Substring matching shows substantial sequence overlap of tryptone peptides and reported bioactive peptides. Establishing sequence overlays between tryptone peptides and 1172 bioactive peptides derived from milk (675) and other sources (510). If a peptide was found in multiple sources, it was counted more than once (exclusively milk: 662; (milk and) other sources: 510).

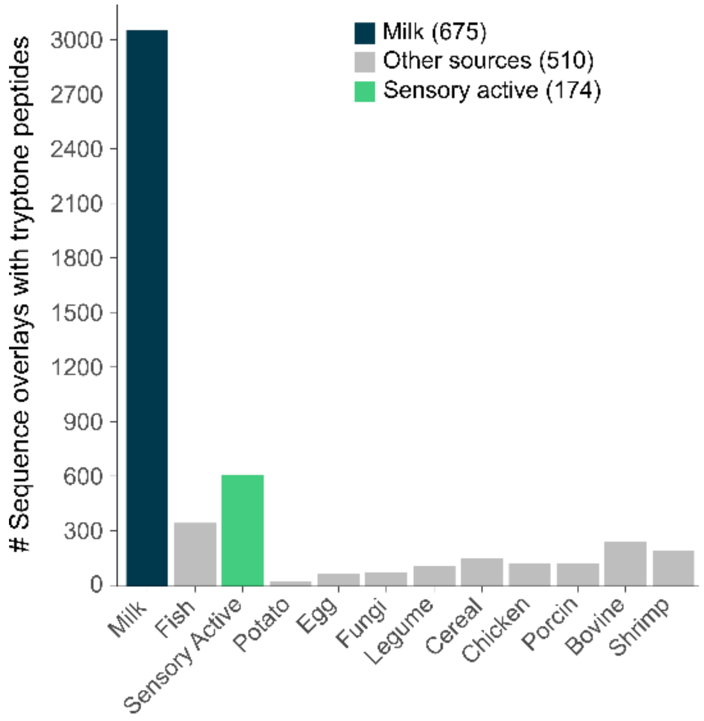

Supplementary Figure 13. Mapping tryptone peptides to established bioactive peptides exposes considerable sequence commonalities with various sources. Number of subsequence overlays between tryptone peptides and peptides that were previously established to be bioactive. Colors indicate different peptide sources.

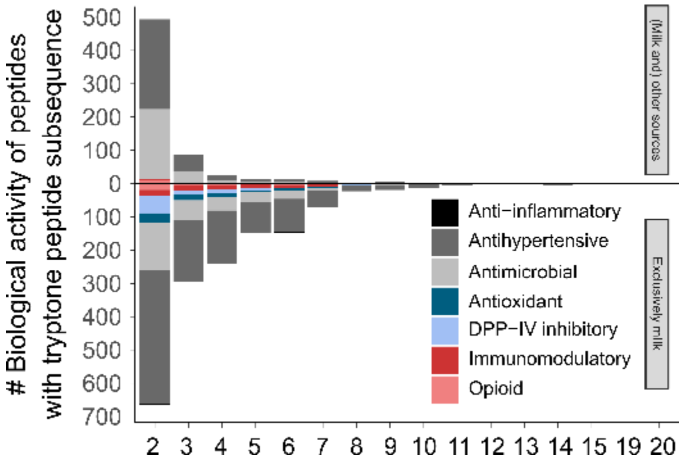

Supplementary Figure 14. Peptides with miscellaneous bioactivities show common subsequences with tryptone peptides. The bar graph compares bioactivity heterogeneity for all the bioactive peptides containing tryptone peptides as a subsequence (exclusively milk: bottom, (also) other sources: top).
